# Supplementary material for: Genome-Wide Analysis of Abscisic Acid Biosynthesis, Catabolism, and Signaling in Sorghum Bicolor under Saline-Alkali Stress
Source: Biomolecules. 2019 Dec 3;9(12):823. doi: 10.3390/biom9120823 (PMC6995594; doi:10.3390/biom9120823)
Supplement: Supplementary file 1 [file biomolecules-09-00823-s001.zip › supplementals/Figure S1.pdf]

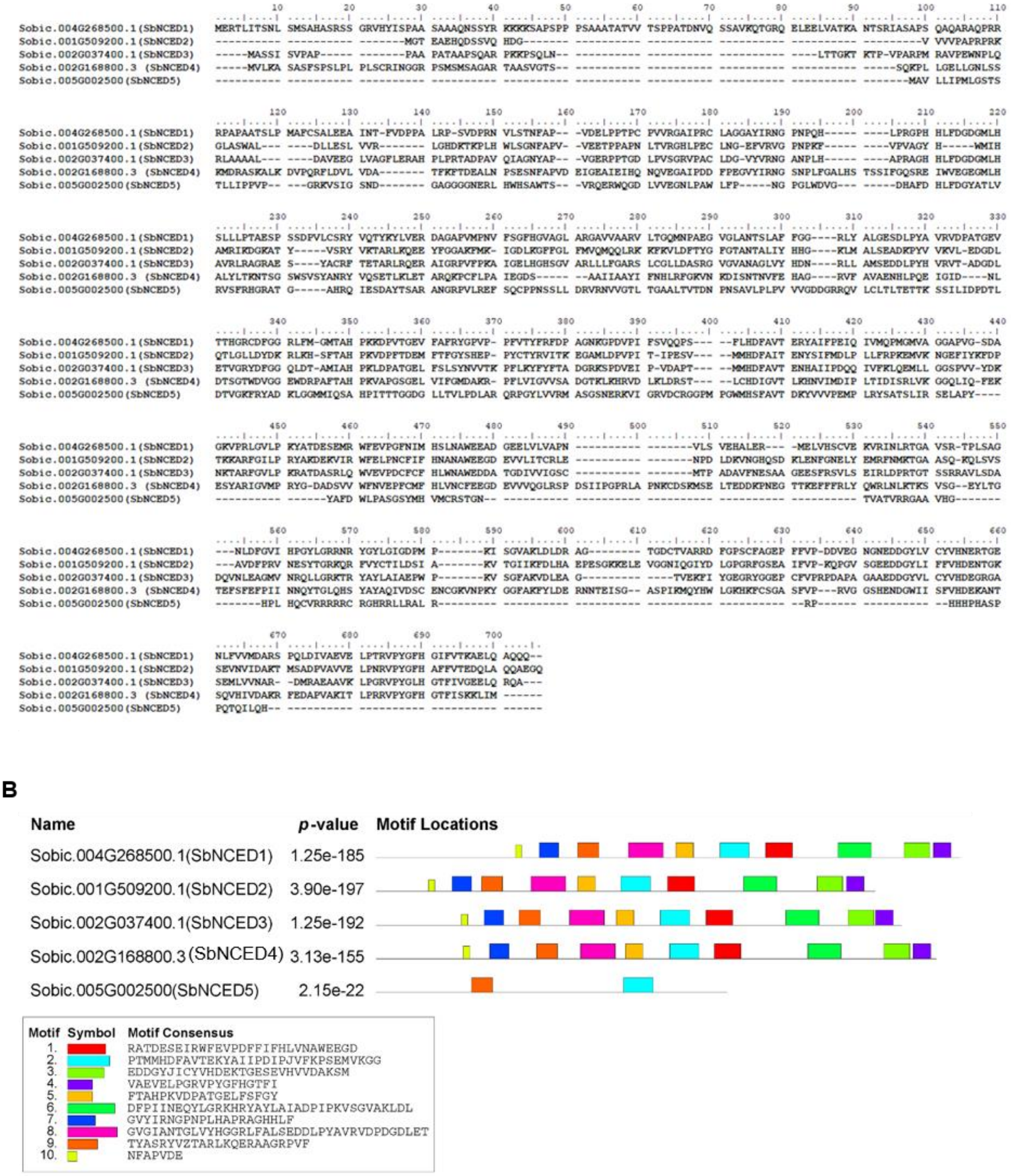

Figure S1 Analysis of the sorghum NCEDs amino acid sequences. (**A**) NCEDs protein sequence alignments. (**B**) Motif distribution among SbNCEDs.
