# Supplementary material for: Genome-Wide Analysis of Abscisic Acid Biosynthesis, Catabolism, and Signaling in Sorghum Bicolor under Saline-Alkali Stress
Source: Biomolecules. 2019 Dec 3;9(12):823. doi: 10.3390/biom9120823 (PMC6995594; doi:10.3390/biom9120823)
Supplement: Supplementary file 1 [file biomolecules-09-00823-s001.zip › supplementals/Supplemental Data Set 1. Protein sequences in this study.doc]

>Sobic.004G268700.1(SbABA8OX1)

MGALLLFVCLLAPIVLLCAAVRGSRKRRSASPASSCGKALPLPPGSMGWPYVGETFQLYSSKNPNVFFARKQNRYGPIFKTHILGCPCVMVSSPEAARFVLVTQAHLFKPTFPASKERMLGPQAIFFQQGDYHTHLRRLVSRAFSPEAIRGSVPAIEAVALRSLDSWDGQLVNTFQEMKLYALNVALLSIFGEEEMRYIEELKQCYLTLEKGYNSMPVNLPGTLFHKAMKARKRLGAIVAHIIEARRGRQQQQQQQQRGRDLLASFLDDREALTDAQIADNVIGVIFAARDTTASVLTWMVKFLGDNPAVLKAVIEEQQEIARSKGSSSDEPLTWADTRRMRMTSRVIQETMRVASILSFTFREAVEDVEYQGYLIPKGWKVMPLFRNIHHSPDHFPCPEKFDPSRFEVAPKPNTFMPFGNGTHSCPGNELAKLEMLVLFHHLATKYRWSTSKSESGVQFGPFALPLNGLPMTFLRKD

>Sobic.002G225400.1(SbABA8OX2)

MAFFFILACTLIFLAIAYYFQYARWQKGRGRSGGGHEKQASSSLKSLPPGSMGWPYLGETLQLYSQDPNIFFASKQKRYGEIFKTHLLGYPCVMLASPEAARFVLVTQAHLFKPTYPRSKERMIGPSALFFHQGDYHLRIRKLVQGALGPDALRALVPEVEAAVRSTLASWDGHVRSTFHAMKTLSFDVGIVMIFGGGRLDERRKAELRKNYSIVEKGYNSFPNSLPGTLYYKAMQARRRLQGVLSDIMRERRERGEPGTDLLGCLMQSQGDDGAPLLSDEQVADNIIGVLFAAQDTTASVLTWIVKYLHDHPKLLEAVRAEQAAVREATDGGRLPLTWAHTRSMALTHRVILESLRMASIISFTFREAVADVEYKGFLIPKGWKVMPLFRSIHHSPDYFQDPHKFDPSRFQVAPRPNTFLPFGNGVHACPGNELAKLEMLVLIHHLVTAYRWEIVGSSDEVEYSPFPVPKHGLPVKLWRENSTVDRKGRQTDDDDVEDIIV

>Sobic.004G268500.1(SbNCED1)

MERTLITSNLSMSAHASRSSGRVHYISPAASAAAQNSSYRKKKKSAPSPPPSAAATATVVTSPPATDNVQSSAVKQTGRQELEELVATKANTSRIASAPSQAQARAQPRRRPAPAATSLPMAFCSALEEAINTFVDPPALRPSVDPRNVLSTNFAPVDELPPTPCPVVRGAIPRCLAGGAYIRNGPNPQHLPRGPHHLFDGDGMLHSLLLPTAESPSSDPVLCSRYVQTYKYLVERDAGAPVMPNVFSGFHGVAGLARGAVVAARVLTGQMNPAEGVGLANTSLAFFGGRLYALGESDLPYAVRVDPATGEVTTHGRCDFGGRLFMGMTAHPKKDPVTGEVFAFRYGPVPPFVTYFRFDPAGNKGPDVPIFSVQQPSFLHDFAVTERYAIFPEIQIVMQPMGMVAGGAPVGSDAGKVPRLGVLPKYATDESEMRWFEVPGFNIMHSLNAWEEADGEELVLVAPNVLSVEHALERMELVHSCVEKVRINLRTGAVSRTPLSAGNLDFGVIHPGYLGRRNRYGYLGIGDPMPKISGVAKLDLDRAGTGDCTVARRDFGPSCFAGEPFFVPDDVEGNGNEDDGYLVCYVHNERTGENLFVVMDARSPQLDIVAEVELPTRVPYGFHGIFVTKAELQAQQQ

>Sobic.001G509200.1(SbNCED2)
MGTEAEHQDSSVQHDGVVVVPAPRPRKGLASWALDLLESLVVRLGHDKTKPLHWLSGNFAPVVEETPPAPNLTVRGHLPECLNGEFVRVGPNPKFVPVAGYHWMIHAMRIKDGKATYVSRYVKTARLKQEEYFGGAKFMKIGDLKGFFGLFMVQMQQLRKKFKVLDFTYGFGTANTALIYHHGKLMALSEADKPYVVKVLEDGDLQTLGLLDYDKRLKHSFTAHPKVDPFTDEMFTFGYSHEPPYCTYRVITKEGAMLDPVPITIPESVMMHDFAITENYSIFMDLPLLFRPKEMVKNGEFIYKFDPTKKARFGILPRYAKDEKVIRWFELPNCFIFHNANAWEEGDEVVLITCRLENPDLDKVNGHQSDKLENFGNELYEMRFNMKTGAASQKQLSVSAVDFPRVNESYTGRKQRFVYCTILDSIAKVTGIIKFDLHAEPESGKKELEVGGNIQGIYDLGPGRFGSEAIFVPKQPGVSGEEDDGYLIFFVHDENTGKSEVNVIDAKTMSADPVAVVELPNRVPYGFHAFFVTEDQLAQQAEGQ

>Sobic.002G037400.1(SbNCED3)

MASSISVPAPPAAPATAAPSQARPKKPSQLNLTTGKTKTPVPARPMRAVPEWNPLQRLAAAALDAVEEGLVAGFLERAHPLPRTADPAVQIAGNYAPVGERPPTGDLPVSGRVPACLDGVYVRNGANPLHAPRAGHHLFDGDGMLHAVRLRAGRAESYACRFTETARLRQERAIGRPVFPKAIGELHGHSGVARLLLFGARSLCGLLDASRGVGVANAGLVYHDNRLLAMSEDDLPYHVRVTADGDLETVGRYDFGGQLDTAMIAHPKLDPATGELFSLSYNVVTKPFLKYFYFTADGRKSPDVEIPVDAPTMMHDFAVTENHAIIPDQQIVFKLQEMLLGGSPVVYDKNKTARFGVLPKRATDASRLQWVEVPDCFCFHLWNAWEDDATGDIVVIGSCMTPADAVFNESAAGEESFRSVLSEIRLDPRTGTSSRRAVLSDADQVNLEAGMVNRQLLGRKTRYAYLAIAEPWPKVSGFAKVDLEAGTVEKFIYGEGRYGGEPCFVPRPDAPAGAAEDDGYVLCYVHDEGRGASEMLVVNARDMRAEAAVKLPGRVPYGLHGTFIVGEELQRQA

>Sobic.002G168800.3 (SbNCED4)

MVLKASASFSPSLPLPLSCRINGGRPSMSMSAGARTAASVGTSSQKPLLGELLGNLSSKMDRASKALKDVPQRFLDVLVDATFKFTDEALNPSESNFAPVDEIGEAIEIHQNQVEGAIPDDFPEGVYIRNGSNPLFGALHSTSSIFGQSREIWVEGEGMLHALYLTKNTSGSWSVSYANRYVQSETLKLETARQKPCFLPAIEGDSAAIIAAYIFNHLRFGKVNKDISNTNVFEHAGRVFAVAENHLPQEIGIDNLDTSGTWDVGGEWDRPAFTAHPKVAPGSGELVIFGMDAKRPFLVIGVVSADGTKLKHRVDLKLDRSTLCHDIGVTLKHNVIMDIPLTIDISRLVKGGQLIQFEKESYARIGVMPRYGDADSVVWFNVEPFCMFHLVNCFEEGDEVVVQGLRSPDSIIPGPRLAPNKCDSKMSELTEDDKPNEGTTKEFFFRLYQWRLNLKTKSVSGEYLTGTEFSFEFPIINNQYTGLQHSYAYAQIVDSCENCGKVNPKYGGFAKFYLDERNNTEISGASPIKMQYHWLGKHKFCSGASFVPRVGGSHENDGWIISFVHDEKANTSQVHIVDAKRFEDAPVAKITLPRRVPYGFHGTFISKKLIM

> Sobic.005G002500(SbNCED5)

MAVLLIPMLGSTSTLLIPPVPGRKVSIGSNDGAGGGGNERLHWHSAWTSVRQERWQGDLVVEGNLPAWLFPNGPGLWDVGDHAFDHLFDGYATLVRVSFRHGRATGAHRQIESDAYTSARANGRPVLREFSQCPPNSSLLDRVRNVVGTLTGAALTVTDNPNSAVLPLPVVVGDDGRRQVLCLTLTETTKSSILIDPDTLDTVGKFRYADKLGGMMIQSAHPITTTGGDGLLTVLPDLARQRPGYLVVRMASGSNERKVIGRVDCRGGPMPGWMHSFAVTDKYVVVPEMPLRYSATSLIRSELAPYYAFDWLPASGSYMHVMCRSTGNTVATVRRGAAVHGHPLHQCVRRRRRCRGHRRLLRALRRPHHHPHASPPQTQILQH

>Sobic.001G289100(SbPYL1)

MVESPNPNSPSRPLCIKYTRAPARHFSPPLPFSSLIISANPIEPKAMDKQGAGGDVEVPAGLGLTAAEYEQLRSTVDAHHRYAVGEGQCSSLLAQRIQAPPAAVWAIVRRFDCPQVYKHFIRSCALRPDPEAGDALRPGRLREVSVISGLPASTSTERLDLLDDAARVFGFSITGGEHRLRNYRSVTTVSELADPGICTVVLESYVVDVPDGNTEDDTRLFADTVIRLNLQKLKSVAEANAAAAASFVSVVPPPEPEE

>Sobic.010G169100(SbPYL2)

METHVERALRATLTEAEVRALEPAVREHHTFPAGRVAAGTTTPTPTTCTSLVAQRVSAPVRAVWPIVRSFGNPQRYKHFVRTCALAAGDGASVGSVREVTVVSGLPASSSTERLEVLDDDRHILSFRVVGGDHRLRNYRSVTSVTEFQPGPYCVVVESYAVDVPEGNTAEDTRMFTDTVVRLNLQKLAAVAEESAAAAAAGNRR

>Sobic.004G097800(SbPYL3)

MEPHMETALRQGGLSELEQRELEPVVRAHHTFPGRSPGTTCTSLVTQRVDAPLSAVWPIVRGFAAPQRYKHFIKSCDLRSGDGATVGSVREVTVVSGLPASTSTERLEILDDDRHILSFRVVGGDHRLRNYRSVTSVTEFHHHHQAAAGRPYCVVVESYVVDVPEGNTEEDTRMFTDTVVKLNLQKLAAIATSSAAAAASNSST

>Sobic.009G170700(SbPYL4)

MPYTAPRPSPQQHSRVTGGGAKAAIVAASHGASCAAVPAEVARHHEHAARAGQCCSAVVQAIAAPVGAVWSVVRRFDRPQAYKHFIRSCRLVDDGGGGAGAGAGATVAVGSVREVRVVSGLPATSSRERLEILDDERRVLSFRVVGGEHRLANYRSVTTVHEAEAGAGGTVVVESYVVDVPPGNTADETRVFVDTIVRCNLQSLARTAERLALALA

>Sobic.001G403300(SbPYL5)

MPCLQASSSPGSMPHQHHGRVLAGVGCAAEVAAAAVAATSPAAGMRCGAHDGEVPAEAARHHEHAAPGPGRCCSAVVQHVAAPASAVWSVVRRFDQPQAYKRFVRSCALLAGDGGVGTLREVRVVSGLPAASSRERLEVLDDESHVLSFRVVGGEHRLQNYLSVTTVHPSPAAPDAATVVVESYVVDVPPGNTPEDTRVFVDTIVKCNLQSLATTAEKLAAV

>Sobic.004G113800 (SbPYL6)

CGCCGGCCCCGCGACGGTGGCGCCTGGCGGACGAGCTGCGGTGCGACCTG

MVEMDGGVGVVGGGQQTPAPRRWRLADELRCDLRAMETDYVRRFHRHEPRDHQCSSAVAKHIKAPVHLVWSLVRRFDQPQLFKPFVSRCEMKGNIEIGSVREVNVKSGLPATRSTERLELLDDNEHILSVKFVGGDHRLQNYSSILTVHPEVIDGRPGTLVIESFVVDVPDGNTKDETCYFVEALLKCNLKSLAEVSERQVIKDQTEPLDR

Sobic.009G080200(SbPYL7)

MVGLVGGSTARAEHVVANAGGETEYVRRLHRHAPAEHQCTSTLVKHIKAPVHLVWELVRSFDQPQRYKPFVRNCVVRGDQLEVGSVRDVNVKTGLPATTSTERLEQLDDDLHILGVKFVGGDHRLQNYSSIITVHPESIDGRPGTLVIESFVVDVPDGNTKDETCYFVEAVIKCNLKSLAEVSEQLAVEPPTSPIDQ-

>Sobic.001G025700.1(SbPP2C01)

MLGPLLRLLSACGGVWPTSPAPGAAGAAASASSSSGDDSEGRDGLLWWRDLARCHAGDVSVAVAQANQVLEDQCRLDSAPPLGTVVGVFDGHGGPDAARFACDHLVPNLREASSGPRGVTADAIREAFLATEEGFLALVSSLWEAQPDIATAGTCCLVGVVHNRTLFVANLGDSRAVLGKKVGRAGQITAEQLCSEHNANQEAVRQELKAQHPDDAQIVALKHGVWRVRGLIQVSRSIGDVYLKHAKYNTERIKPKFRLSESFSKPLLSADPAIISRNLEPNDCFIIFASDGLWEHLSNQEAVEIVHNHQHAGSARRLIKAALQEAARKREMRYSDLTKIDKKVRRHFHDDITVIVLFINHDLLLKGAPQGQPLSIRCALDY

>Sobic.001G034100.1(SbPP2C02)

MGNSLACFCCAGGAAGRRRHVAPAALPSDPAYDEGLGHSFCYVRPDKVLVPFSADDDLVADAKAAAAAEEATTFRAISGAALSANVSTPLSTSVLLLLPDDSTASSAAAASSGFESSESFAAVPLQPVPRFPSGPICAPAGGGFLSGPIERGFLSGPLDAALMSGPLPGAATSGRMGGAVPALRRSLSHGGRRLRNFTRALLARAEKLHDSLDLGSPDAAAAVAACGGGSAGLQWAQGKAGEDRVHIVVSEERGWVFVGIYDGFNGPDATDFLVTHLYAAVHRELRGLLWDQCEREEQHDTHPDQPTSTTASDHPDQPPNRRRARRSRPPRGADDDQRRWKCEWERDCSSLKPPTQRPARSSSENDHLAVLKALARALRKTEEAYLDVADKMVGEFPELALMGSCVLAMLMKGEDMYLMNVGDSRAVLGTMDSVDLEQISEGSFDGLVGDGTPLLSAVQLTSEHSTSVREEVCRIRNEHPDDPSAISKDRVKGSLKVTRAFGAGYLKQPKWNDALLEMFRIDYVGSSPYITCNPSLFHHRLSTRDRFLILSSDGLYQYFTNEEAVAQVEMFIATTPEGDPAQHLVEEVLFRAANKAGMDFHELIEIPHGDRRRYHDDVSVIVISLEGRIWRSCV

>Sobic.001G081600.1(SbPP2C03)

MLRAVARCCGHWPPGAAAADGMLWQTELRPHAAGEFSMAAAQANVTMEDQAQVLASPAATLVGVYDGHGGADASRFLRSRLFPHVQRFAREQGGMSAEAIRSAFGAAEEEFHKQVRQEWTKRPRLAAVGSCCLLGAISGDTLYVANAGDSRAVLGRRVVGGGVAVAERLSTEHNAASEEVRRELTALNPDDAQIVVHARGAWRVKGIIQVSRSIGDFYLKKPEYSLDPLFRQVGAPIPLKRPALSAEPSVQVRKLKPNDLFLIFASDGLWEHLSDDDAVQIVFKNPRTGIANRLVKAALKEATRKREVRYRDLRTIDRGVRRHFHDDISVVVVYLDRHRERRHTRVIDSSSNCTSAPVDIYSSNTHQSAEPLHAYKC

>Sobic.001G309500.1(SbPP2C04)

MDYLKSCWGPASPAGRPRRGSDATGRQDGLLWYKDGGQVVDGEFSMAVVQANNLLEDHSQVESGPLSTSEPGLQGTFVGVYDGHGGPETARYINDHLFNHLRRFASEHKGMSADVIRKAFRATEEGFISVVSDQWSVRPQLAAVGSCCLVGVVCSGTLYVANLGDSRAVLGRLVKGTGEVLAMQLSAEHNASYEEVRRELQASHPDDPHIVVLKHNVWRVKGIIQITRSIGDAYLKKPEFNREPLHSKFRLQETFRRPLLSADPAITVHQIQPTDKFIIFASDGLWEHLSNQEAVDMVQSSPRNGIARKLVKSAMQEAAKKREMRYSDLKKIDRGVRRHFHDDITVIVVFFDSNAMTAAAWSRPSVSLRGGGVPIPSNTLAPFSVPTELNNSY

>Sobic.001G312500.1(SbPP2C05)

MSGGVEPETPPGSSSRSGGSTPVGGKPPRHHLTSIRHCASSARIAAASAEFGLDSGTLSLISPPADIRPGFLPVFRSGSFADIGPKSYMEDEHVCVDNLVEHLGLRVPGIPAPGAFYGVFDGHGGADAACFVRKNILKFITEDCHFPNSIEKAIRSAFVKADHAIADSQSLDRNSGTTALTVLISGRTLLVANAGDCRAVLGKRGRAVELSRDHKPSCTVERLRIENLGGTVFDGYLNGQLAVARAIGDWHMKGSKGSACPLTPEPEFREVRLTEEDEFLIIGCDGLWDVMSSQFAVSMVRKELMEHNDPQRCSRELVQEALRRDCCDNLTVVVVCFSADPPPQIEVPRFRVRRSISMEGLHTLKGALDSNV

>Sobic.001G358800.1(SbPP2C06)

MDSETRSGSFRHEPAAADDLAAAAAGLQRPCRSFGETTFRTISGASVSANASSARTGNLTVSLLAGDVQEPAAAFESTASFAAVPLQPVPRGSGPLNTFLSGPLERGFASGPLDKGSGFMSGPLDKGAFMSGPIDGGSRSNFSAPLSYGRRKARLGLLVHRISRPMKTALSRTFSRSSHNPGWVQKFLMHPMAQLPWARDAKSRSEGSQNGLEPGIPEPEYNVTRNLQWAHGKAGEDRVHVVLSEEQGWLFIGIYDGFSGPDAPDFLMSNLYKAIDKELEGLLWVYEDSSERCDHVSNLEEGESASASVDAPHDDNGQFQSDNGKQEQLGNFGKQNVSPGKGCDESVLQVQPNCTSCEEKDLAAQDSSSQKLGRDEIVEEMAGADLGNDLQSRESHNSNSGLSGTDLNTSCTCATETSAYCDQHAKFLKENRKSKRLFELLEMELLQDYNKRLSKASLEERKIPSLHVAQAGTAEGSSRNAAEISRCSLAATGECFDDSEDLGSSRHADSVLGTDLKECTGCSISTSSSEHKQVTRRFVFGSKLRKMYKKQKMLQKKFFPWNYDWHRDQPHVDESVIKSSEVTRRCKSGPVEHDAVLRAMSRALETTEEAYMEIVEKELDRHPELALMGSCVLVMLMKDQDVYVMNLGDSRAILAQDNDQYNSSSFLKGDLRHRNRSRESLVRVELDRISEESPMHNPNSHLNSNTKAKELSICRLKMRAVQLSTDHSTSIEEEVLRIKVEHPDDPQAVFNDRVKGQLKVTRAFGAGFLKKPKFNEALLEMFRIDYVGTSPYISCNPAVLHHRLCANDRFLVLSSDGLYQYFSNDEVVSHVLWFMENVPEGDPAQYLVAELLCRAAKKNGMNFHELLDIPQGDRRKYHDDVSVMVISLEGRIWRSSG

>Sobic.001G399300.1(SbPP2C07)

MAALAASEGNLSPTLPLATLIGRELRGDGTERPHVRYGHSGFAKRGEDYFLVKPDCLRVPGDPSSSFSVFAVFDGHNGVSAAVFSKEKLLEHVMSAVPQGISREDWLQALPRALVAGFVKTDIDFQRKGETSGTTATLVVVDGFTVTVASVGDSRCILDTHGGEVSLLTVDHRLEENAEERERVTASGGEVSRLNLCGGQEVGPLRCWPGGLCLSRSIGDTDVGEFIVPIPHVKQVKLPNTGGRLIIASDGIWDALSSEIAAQACRGLPAELAAKLVVKQALKTSGLKDDTTCVVVDIIPSDHCSTPPALSPKKNQNKLRSLIFGRRSHSSVGKLTKSASLGSVEEIFEEGSAMLEERLGRNFPSKANLPPFRCAICQVDQEPFEGLMTDNVGGCCSAPSTPWGGPYLCSDCRKKKDAMEGKRSNRSATCR

>Sobic.001G406300.1(SbPP2C08)

MSCTVAIPSSPVFSPSRRPLSCKAASASPEPAVAVSASSPAPAPAAAAGSPLRPFALRALLREEVSPSPSPQSASAAAVATAPTGAVLKRRRPAPLVVPASGAAAAAAAAAAVAAVEADPRNEVEEEGEEFAAYCRRGKGRRRVEMEDRHVAKVALGGDPQVALFGVFDGHGGKNAAEFAAENMPKFMAEEFKKVNGGEIEGAVKRGYLKTDEEFLKRDESGGACCVTAVLQKGGLVVSNAGDCRAVLSRAGKAEALTSDHRASREDEKERIENLGGFVVNYRGTWRVQGSLAVSRGIGDGHLKQWVVADPDTRTVLVDQQCEFLILASDGLWDKIDNQEAVDLARPLCINNDKTSRMAACRMLTETSISRGSTDDISVVIVQLQNFSSS

>Sobic.001G424400.1(SbPP2C09)

MAEICCEVAATGSDRKGECVGGDAGSRAARKRRMEIRRLRVVAEEASAKRRRLEADEEDAVRRPAPRYGVTSVCGRRRDMEDAVTTRLGFIDGHHFFGVFDGHGCSHVATSCGQRMHQIVAEEATAAAGSSASDDAARWRDVMEKSYSRMDAEAVGSRDTAGPAPTCRCEMQLPKCDHVGSTAVVAVVGPRHLVVANCGDSRAVLCSGGAAIPLSDDHKPDRPDELERIHAAGGRVIFWDGARVFGMLAMSRAIGDSYLKPFVISDPEVRVVERKDGEDEFLILASDGLWDVVSNEVACKVVRTCLRNRAPPHPHGGDGERSSPTSNLSPRQSSGSGSSSGDEEGAGPSDGAGSESDGDGESSEDRACSEAAILLTKLALARQTADNVSVVVVNLRRRRRS

>Sobic.001G462800.1(SbPP2C10)

MRGLRRWLPFCGGGCCCCGEGGAGRGGGVADGLVWDVALKAHASGDYSIAVAQANESLEDQAQVLAAPAATLVGVFDGHGGPEAARFVNRRIFSHIQGFAAENGGLSAEVLQKAFGATEEEFIGLVQKSWPSQPRIVSVGSCCLVGAIEGGTLYVANLGDSRAVLGRRGGGGKGNRRVVAERLSQDHNVADEDVRREVAEMHPDEPHIVLNSHGVWRIKGIIQVSRSIGDVYLKKPDICRNNPALQQSLCPFPLRRPVMSAVPSITTRELRPGDRFLIFASDGLWEQLSDEAAVGVVASSPRKGVAMRLVRAAQLEAARKKEVKYDKIRTIEKGQRRHFHDDITVVVLFLDKCRGAARSGPEDIDGTYAPLDVFSYSPAGDHEDPTKPVLRR

>Sobic.001G512600.1(SbPP2C11)

MLAAVMDYFSSCWGPRSRAGHRGKGSDAAGRQDGLLWYKDAGQLVTGEFSMAVVQANQLLEDQSQVESGPLSLADPGPQGTFVGVHDGHGGPETARFINDHLFNHLRKFATEHKCVSADVIRKAFQATEEGWLSLVSKEWSMKPQIASVGSCCLVGVICAGTLYVANLGDSRAVLGRLVKATGEAVAMQLSSEHNACHEEVRQELQSSHPDDPHIVVLKHNVWRVKGLIQISRSIGDVYLKKPEYNREPLHSKFRLRETFQRPILSADPQITEHRIQPNDQFVIFASDGLWEHLSTQEAVDLVHSSPRNGIARRLVKAAMQEAAKKREMRYSDLKKIDRGVRRHFHDDTTVVVLFLDSNAMSKASWSKSPSVSIRGGGVTLPAKSLAPFSALAQLNSTY

>Sobic.002G076500.1(SbPP2C12)

MAAPPLVFVFAAIAALAILVLVVFAFRRWWRRRGQRQRQRRPLQTAEAAPTPVAVQDEDIDRPLLSGNWGDHSRQSNSFLGSSVGEPSKIQTNRSNTSPRSHAITDTGRIYPAECCATQGETHVINVENDTTEEYQLGSTLKRTPPPKWSTPDQKHRRRVSGEDNHNGSVSLKDNTYHSSLNLEVIAGPSHGISCSRQSSRPSMLPITLGRVPPSDLVFKDSEVSGKHAQINWNAKTLKWELVDMGSLNGTFLNSQSVHHPDAQSRRWGEPAELAHGDIITLGTSSKLSVQISLQNQRVPAGVGMASDPMVARRSGRKLPMEDISFCQCPLQGVEHFGFFGIFDGHGGDGAAKAVSKILPENLGYILSHPETKERVQSCADASDVLRYAFTLTEDAIDHQYEGCTGTALLIWFDQNKDCFAQCANLGDSACVMSVNGRTIDMTEDHRVASATERARIARTGQPLKDGEVRLNGLNLARMFGDKFLKEQDSRFSSEPYVSQAVRITKACTAFAVIASDGLWDVISTKKAVQLVVEGKERNAGDSTSAAKVANRVLNEARNLRTKDNTSVIFVDFDILRTDLCIAK

>Sobic.002G172000.1(SbPP2C13)

MSMAQVCCDSAAAATAAAAQVVGAEAEARARARAERRRRAGEAAGRWRHAGAAAGGTPDAATRKRSLEAGELLVARKHGAASVAGRRREMEDAVSVREAFALAEGSHGGRRDFYGVFDGHGCSHVAEACRDRMHELLAEELAVAAAADDVSWTAAMERSFARMDSEVMSAGGASGACGCDAHKCDHVGSTAVVAVVEERRVVVANCGDSRAVLCRGGDGAPPVPLSSDHKPDRPDELARIEAAGGRVIFWEGARVLGVLAMSRAIGDGYLKPYVSSVPEVTVTDRSDGDECLILASDGLWDVVSNEAACEVARACLRRGRAKWCAEAAALLTKLALARRSSDNVSVVVVDLRRRNHH

>Sobic.002G226800.1(SbPP2C14)

MAMAAAAPAALSPGTVPLGVLLRREVTKERMERPDVLCGEANRSRKGEDFTLLRADAGQRIPGDPSTSFSVFALFDGHNGSGAAIYARENLLKNVLRAIPTCFCREEWLAVLPRALVAAFVKTDKDFQAVAETSGTTVTFVIIDEWVVTVASVGDSRCILESADGSIYFLSADHRFDSNPDEVKRVTACGSKVGKMDIVGGPEVGPLRCWPGGLCLSRSIGDLDVGECIIAVPHVKQVKLSNAGGRIVIASDGVWDDLTCEMALDCSRGFSSDVAAHRIVNEATRPRGIRDDTTCIVIDILPPENIAPIPSPPKRPSRIAFNNMFRRRSLDVPFKTNRSEYAEPDVVEEIFEDGSPMLSKRLTTGYTLEKIFAPSSCAVCLVRLKSGEGISVHANPLQHEKLQGWQGPFLCLSCHEKKEAMEGKRRPRGSSTNVFGHMC

>Sobic.002G294400.1(SbPP2C15)

MGVYLSTPKTDKLSADGENSRVRFGLSSMQGWRTTMEDAHAAFPDLDDCTSFFGVYDGHGGKGVSKFCARHLHKQVLINDANSSGDLPASVHKAFLRMDEMMKGQRGWRELTELGDKGNKISGMLEGIIWPPKGGDSDKIEEDWDNEEGPNSNFPGPTSGSTACVAVIRNDQLIVANAGDSRCVISRKGQAYNLSTDHKPDLEGEKERILGAGGFVVAGRVNGSLNLSRAIGDMELKQNELLPAERQIVTAEPELKTVQLSEDDEFIVLACDGIWDCMSSQEVVDFVHKQLKTEDKLSSVCEKMLNRCVAPTSGGEGCDNMTVIVVQFKKPASAVATSSTEQSSATAEEMRPNELDGPDNPPK

>Sobic.002G312900.1(SbPP2C16)

MAGKEILHKVKDKVKDAFSSSGPETGKGKTKLSGKRVKHGYHLVKGKSNHPMEDYLVAEYRQVGEHDLGLFAIFDGHLGHTVPDFLRSHLFNNILSEPEFLSDPETAIRNAYQLTDQKILENAAELGRGGSTAVTAILIGSDKSVKLVVANVGDSRAVISKNGVAKQLSVDHEPNMERQTIEQKGGFVSNLPGDVPRVDGQLAVARAFGDRSLKKHLSSDPYVIEETIDEYTDFLILASDGLWKVMSNQEAVDAIKDCKDAQAAAKHLTEQAVNRKSKDDISVIVVKFLC

>Sobic.002G317500.1(SbPP2C17)

MASLQQRKEESGGDHGHAAADEAVRGTGKGKSEAAPGRRKVTYGFHLAEGRTPHGMEDRHVAEFRQLDDGNEVGLFAGHSGADVATYLREHLFDNILNQPGFDFWTDPMEAIRRAYHRTDRKVLKKKKKKDDDGEGKGMGSRRRGGSTAVTAILMNGEELVVANVGDSRAVLCDAGGRARQLSVDHEPLRERRAIEARGGFVTEIHGDVPRVDAQLAMARAFGDRSLKEHISADPDVAIEDVGDGAELLVLASDGLWKVMSNQEAVDEARSIEDAREAAVRLVDEAVRRGSKDDISCIVVRLH

>Sobic.002G397600.1(SbPP2C18)

MGCAQGKCCLPRHPRRGSSCAVGGRGDHGGATLGRAAVPGAGLVLEYAALAVAGLYPDSPARESQDAHLVATRFAGDPDLHLFAVFDGHGAAGAACAGFARDALLRLLLASPSLAADPAAAFREAMLGANAEMHAAPGVDDSMSGTTAVAALVAGGALHVANVGDSRAVAGVWRAGRVVAEDLSWDQTPFRADERARVKACGARVMSVEQVEGMRDPEAEGWAPDEGDPPRVWARDGLYPGTAFTRSLGDLAAEAVGVVAEPEVKSVEITPSHLFFVVASDGVFEFLSSQEVVDRVATYPDPRDACSAIAAESYKLWLEHENRTDDITIVIVHIRDMENSGPAGSDKADYGSVGASIAVHTIQSEIPVFVQSEASHMNRSAATGMQSPSSGSPTERSLSCVAPSPTHPLLIHGRISDASKPVQSERAATQEVQTWHQMDGGTELEWSMQQSIPPAS

>Sobic.002G427300.1(SbPP2C19)

MATAARDRRLPPALPLATLIGRELRAGGSERPALRYGHAGFAKRGEDYFLVKPDCLRVPGDPASAFSVFAVFDGHNGVSAAVYSKEHLLEHVMSALPPDIGREDWLQALPRALVAGFVKADIDFQRKGEVSGTTATLVVIDGFTVTVASVGDSRCILDTQGGELQLLTVDHRLEENAEERERVTASGGEVGRLNLFGGQEVGPLRCWPGGLCLSRSIGDMDVGEFIVPIPHVKQVKLSNTGGRLIIASDGIWDALSNEAAAQACRGLPAELAAKLVVKQALKTSGLKDDTTCVVVDIIPSDHLTSPQLSPKKNQNKLKSLFRRRSHSSVGKLGGKSASIGSVEELFEEGSAMLEERLGRNLSLKAASPPFRCAICQVDQEPFEDLMTDNGGGYCSSPYAPWGGPHLCLDCRKKKDAMEGKRSSRSTACR

>Sobic.003G141000.1(SbPP2C20)

MGAGAEVMHQVVPLLEPPFHRCVVKSVDVMEEVVTVAPGQVQPATSPKAVVEVAVEVPDLEFKRASNAGSSVSAEQLQFVPSIRSGSFADIGPRRFMEDEHIRIDDLSGHLGSLLMVSAPSAFYGVFDGHGGSDAAAYMKTHAMRLFFEDADFPQTSQEDEIYAGSVEDSVRKAFLRADLALADDSVINRSSGTTALTALVLGRQLLVANVGDCRAVLCRKGTAVEISKDHRPTYDAERQRVIECGGYIEDGYLNGVLSVTRALGDWDMKLPQGSPSPLIAEPEIHWTTLTEEDEFLIIGCDGIWDVMSSQHAVSTVRKGLRRHDDPERCARELAMEAKRLETFDNLTVIIVCFVPDLAGAASAAAPSSEQAPAPAGRIRCCKSLSPEALCKLRRWLESDH

>Sobic.003G182600.1(SbPP2C21)

MTYTEQQLTAAIVSQGLRPALALPESGAPPTLLSLIQRCWDPDPEKRPSFEDIIDELNIVQTHLVSNASVPSSAVSKSQNGTIEVHQYQEALNWLNQGELFVKRSSRSDLKSLWSGCFDQSSEYHPTLSWGSFATCGRRETMEDTHFLLPHMSEEKDVFAFGIFDGHRGSAAAEFSVRAVPGFLKQFGQGASPTDALSEAFVSTDLAFREELILHR

>Sobic.003G185900.1(SbPP2C22)

MALLSPRVPRLPPSTFTFTFAAAAGAPCFGRAARCQATAAGGVAAAGPPSSELEAVQWGSAKLQGVRDEMEDEILLRPGSLLDGFSFAAVLDGHAGFSAVQFLRDELYKECAAALDGGAVLSTKNLDAITVSIQRAFAAVDAKLSTWLEQADKDDDSGATATAMFLRNDVLVVSHIGDSCLQVISRGGRPEALTSSHRPYGNNKTSLEEVKRIRAAGGWIVDGRICGDISVSRAFGDIRFKTRKNEMLVKGVKEGRWTDKFISRIKFKDDLIISSPDVSLVELGPDVEFVLLATDGLWDYIKSSEAVAFVRDQLRQHGDVQLACEALGQKALDQRSKDNISIVIADLGRTNWKALPDERPNMFLELSQAVATVGVVSIGIWFSSFLGLQ

>Sobic.003G198200.1(SbPP2C23)

MEDVVAVVAPLPAPPAPTFSPAAAGLTLIAAAAADPIAAAVVGAMEGVSVPVTVPPVRTASAVDDDALPPEGEGEGGDASVAGSPCSVASDCSSVASADFEGVGLGFFGAEGGPMVFEDSAASAATVEAEARVAAGGRSVFAVDCVPLWGYTSICGRRPEMEDAVAIVPRFFDVPLWMLTGNAVVDGLDPMTFRLPAHFFGVYDGHGGAQVANYCRERLHVALLEQLSRIEENVCAANLVDMEFKKQWEKAFVDSFARVDDEVGGKAIRGGGGEAGTSNAAVALAPEPVAPETVGSTAVVAVICSSHIIVSNCGDSRAVLYRGKQPVPLSVDHKPNREDEYARIEAEGGKVIQWNGYRVFGVLAMSRSIGDRYLKPWIIPVPEVTIVPRAKEDECLILASDGLWDVMSNEEVCEVARKRILLWHKKNGTSSSSAPRVGDSADPAAQAAAECLSKLAVQKGSKDNITVVVVDLKAHRKFKSKT

>Sobic.003G198900.1(SbPP2C24)

MVTVAAGRRAGAAGGVGGRRRSGCGGAGQAAQQNSPLQQKLLAVAVAARFAEAGPPPSTEASGAGAGGCCVELLDCLLGALNVSVATVTPAPAQYGWAVREIRPRGEGSAEDRHADAPSGRIAGNGASANAAASLYTMQGRKGVNQDAMVVWENFGSKDDTVFCGVFDGHGPNGHLVAKRVRDLLPVKLSANLGKGECKEISTSNVKSGTTKRVATEHRVQDTDASTGNEENGEYPEFFTALRASFLKAFYVMDRDLKAHRNIDCAFSGTTAVTVIKQGQNLIIGNLGDSRAVLGTRDEHNHLVALQLTVDLKPSIPSEAARIRQRRGRVFSLPDEPDVARVWLPTFNSPGLAMARSFGDFCLKNYGIISMPDVSYHRITDKDEFVVLATDGVWDVLSNAEVVSIISSAPSQASAARFLVESAQRAWRTRYPTSKTDDCAVVCLFLNTEAASASSSSVTKVW

>Sobic.003G222400.1(SbPP2C25)

MACDGGGVKEQEGPSAASPAPSPAAVARARARAARPPRPRSRKGLGVRNQLKPCRILAALQRANARVREAGEATLVAALAAAQKESERENEVVPDVCGGWKSEDGSLNCGYSSIRGRRATMEDFYDIKSSRIDDKQIKFFGVFDGHGGTRAAGYLKQHLFENLLKHPGFIGDTKSAMSESYKKTDADFLDAEGNIQVGSTASTAVLIDNHLYVANVGDSRAVMSKAGKAIALSDDHKPNRSDEQKRIEDAGGVVVWSGTWRVGGILAMSRAFGNRLLKQFVVADPEIQDLEIDGDVEFLILASDGLWDVVPNEHAVAFVKDEDSPEAAARKLTEIAFRRGSTDNITCIVVEFCHDKMIDGSLPSTNQS

>Sobic.003G242200.1(SbPP2C26)

MSSSSEASTQDHGVTAQAGAEEHGVPALEVQASAEEHVLPALEAQDSGHDHGVPAFDEEPTPADTDRILAAVAQLARRRRLEARRLGRTAPAGAEGEAPKRARPVSAGSSSRESAKVAPEPQEGASEVVPPGGWPKSVSHGGLSVIGRRREMEDAFAVAAPFLLVKAAAGKEEEESGEGEEKEKEKDGGGGEAEAEAEAEVEFFAVYDGHGGPRVADTCRERLHVVLAEEVARLHLQLGKGGGGDDGGGVLRRWREAMEACFARVDGEVVVVEREVNKNKNNAGDTVGCGSTAVVAVVGPRHIVVANCGDSRAVLSRGGVPMPLSSDHKPDRPDELERVESAGGRVINWNGYRVLGVLATSRAIGDYYMKPFISAEPEVTVTERTQKDEFIILASDGLWDVMTNEVACKVARNCLCGRAAAKYPDTVHGSSASDAASMLVEFAMSRGSTDNISVVVVELKRLKRWKGARQNGRT

>Sobic.003G354000.1(SbPP2C27)

MAEICCEEAKSTPATAVAAASVSAAAAAVAVASSALERRRRRLEMRRFRVATDPEAPVAEDVRATKRQRLLARTVSGSCPDAGSDSDRPALPERLPRYGVTSVCGRRREMEDMVSIRPDFLPGTSTQHFFGVFDGHGCSHVATLCQDMMHEVVADEHRKAGCGEETAWKGVMERSFARLDEQAASWATSRSRDEPACRCEQQMPLRCDHVGSTAVVAVVSPTHVVVANAGDSRAVLSRAGVAVPLSVDHKPDRPDELARIEAAGGRVIYWDGARVLGVLAMSRAIGDGYLKPFVSSEPEVTVTERTDDDECLILASDGLWDVVTNEMACEVVRACFRSNGPPQAPAARPNGVAALPAADADDENGPAVVKGVSKEDSDRACSDAAMLLAKLALARRSADNVSVVVVDLRRGI

>Sobic.004G041500.1(SbPP2C28)

MREEGPWGPPPPPAPAPPSARLSLSLVVLLLLAGPSSCWCCQGREGGAEVARMGLAGDGSADTAQHLSNSENGRFSYGVASSPGKRASMEDFYEARIDDVDGEKVGMFGVYDGHGGVRAAEYVKQHLFSNLIKHPKFITDTKAAIAETYNQTDSEFLKADSSQTRDAGSTASTAIIVGDRLLVANVGDSRAVICKGGQAIAVSRDHKPDQTDERQRIEDAGGFVMWAGTWRVGGVLAVSRAFGDKLLKQYVVADPEIKEEVVDSSLEFLILASDGLWDVVTNEEAVAMVKPIQDPQEAANKLLEEASRRGSSDNITVVIVRFLDGTTGDKSGEDKETTNDQNS

>Sobic.004G065200.1(SbPP2C29)

MGVYLSTPKTDKLSENGENDRLKFGLSSMQGWRATMEDAHSALLDLDSETAFFGVFDGHGGRVVAKFCAKYLHGQVLKSEAYSAGDLGAAVHRAFFRMDEMMRGQRGWRELSALGDKINKFSGMIEGLIWSPRGSDSNSQQDDWASEEGPHSDFAGPTCGSTACVALIRNSQLVVANAGDSRCVISRGGKAYNLSRDHKPELAVERERIMKAGGFIHMGRVNGSLNLSRAIGDVELKQNKFLPPEKQIVTANPDINVVELCDDDDFVVVACDGIWDCMSSQQLVDFIHERINMESSLSAVCERVLDRCLAPSTIGGDGCDNMTMILVQIKKPVNRNKKAEVAGQSANNADEVKSRVAEE

>Sobic.004G143700.1(SbPP2C30)

MVEAAAGRRPGTSRRRPSGSGGEHQRLVALAVAARVAMVTTRSAGPAVAGGDGAGTAGTGAGAAGGSGRCMEDFFDCLLGLLGALGMTWAAARPQRQPRPPLPRGVGAGAVPTDARRFAAELRATPGRIAGNGACTVASLYTLQGKKGVNQDAMIVWENFCSRDDTIFCGVFDGHGPYGHLVAKRVRDLLPLKLGADLGTEDGRVTSTGNIKLNTHDVASPEHKDRGGTAISSETQQNGEYPEIFPALRTSFLKAFHVMDRDLKLHKNIDCFFSGTTAVAVIKQGRNLIIGNLGDSRAVLGTRDENNELVAVQLTVDLKPNIPSEAQRIRQRRGRIFALPEEPEVARVWLPKYNSPGLAMARAFGDFCLKDHGVISMPDVSYHHITEKDEFVVLATDGVWDVLSNDEVVSTVSQATSRASAARFLVETAHRAWRTRFPTSKIDDCAVVCLFLNTNEASESSSSLANNLADAVEVSSAQRSTTIQVSTGASIDVTALVTDGNEESVVETVTRPVTLMDLPKDG

>Sobic.004G186300.1(SbPP2C31)

MSSRSGSTSGGSSQGGGAAVPLAVLLRREVASERTASERPELHSGLFSQAKKGEDFTFLKPECERVPGVPSSSFSAFGLFDGHNGNGAAIYTKENLLNNILSAVPADLNREDWLAALPRALVAAFVKTDKDFQTKARSSGTTVTFVIIDGLVITVASVGDSRCVLQAEGSIYHLSSDHRFDASKEEVDRVTECGGDVGRLNVVGGAEIGPLRCWPGGLCLSRSIGDQDVGEFIVPVPLVKQVKLSTAGGRLIIASDGVWDALSPEVAFNCSRELPPVPAAEQIVKTAVQSKGLRDDTTCIVVDIIAEKNHPSMPHPKKQPGMGVFKNMFGKKKSSDSSSHSHTDREYMDPDNVEEIFEDGCALLSRRLDSEYPVRNMFKLFICAICQVELKPNQGISVHEDSSQPGSLRRWDGPFLCQSCQEKKEAMEGKRRSRDSSRNSGSSE

>Sobic.004G275900.1(SbPP2C32)

MGSGASRLLTACACSRPAPAPADDGPCLDDALGHSFCYAASSAAAAGHSSSFRHAISGAALSANSSVPVPIYHSSGGAGGMPPPQYSSAFHTSSSFSSAPLQLSNLSSGPLFLSGPIDRGAQLSGPLDQAVPFSGPLPAKPTKPAPSSSSRGFSRRFRKPSFGSLRRSVSEKNRPCVVPLRREDGVQWAHGRAGEDRVHVVVSEDQRWLFVGIYDGFNGPEAPDFLVANLYRFLLRELRGIFYEEADPDSKRLWQFLADGDDEDSELDFSGSGRFALSLARLKEQRHPLWAHAAAVGDGQSGREWGVKRLTAAPAVRDHMAVLSALARALATTESAYLDMTSQSMGSHPELAVTGACLLVALLRDDDVYVMNLGDSRAIVAQRRDDDDCLIGSMRVEDIGVGLETESRIPGYSAIGLEALQLSTDHSTSIEGEVQRIRREHPDDDQCIVNDRVKGRLKVTRAFGAGYLKQAKLNDGLLEMFRNEYIGDTPYISCTPSLCHHKLSARDQFLVLSSDGLYQYLSNEEVVLHVENFMERFPEGDPAQSLIEELLSRAAKKAGMDFYELLDIPQGDRRKYHDDVTIMVISLEGRIWKSSGTYV

>Sobic.004G279200.1(SbPP2C33)

MWSWLTKIASACLGPVRRCARTRKGEDGASDNGRGVADDLLWSRDLGRHAAGEFSFAVVQANEALEDHSQVETGSAATFVGVYDGHGGAEAARFISDHIFAHLIRLAQENGTISEDVVRSAVSATEEDFLTLVRRTHFIKPLIAAVGSCCLVGVIWRGTLYVANLGDSRAVIGCLGRSNKIMAEPLTRDHNASMEEVRQELISRHPDDSQIVVLKHGVWRIKGIIQVSRTIGDAYLKRREFALDPSITRFRLSEPLRRPVLTAEPSMCTRVLNPQDKFIIFASDGLWEQLTNQQAVEIVHSNPRRGIAKRLIRAALKQAARKREMRYNDLRKVEKGVRRFFHDDITVVVVYIEHGLLQGRDTSVPELSVRGFVDSVGPSSFSGITAIS

>Sobic.004G332900.1(SbPP2C34)

MCVEELDDAAGRLGFGDQEIDRRAGAAMERVCENTVSVEFKQTKLNNFVPFIRSGDWSDIGSRDYMEDAHVCISDLAKNFGYNSVDDEVISFYGVFDGHNGKDAAHYVRDNLPRVIVEDADFPLELEKVVKRSFVQTDSKFAEKFSHQKGLSSGTTALTAMIFGRSLLVANAGDCRAVLSRRGTAMEMSKDHRPCCINERKRVESLGGYVDDGYLNGQLAVTRALGDWHLEGMKEMGEPGGPLSAEPELKMVTLTKDDEFLIIGSDGIWDFFSSQNAVDFARRKLQDHNDLRLCCREIVEEAIRRGARDNLTAVMVSFHQDAPPQSRWNRTREGSVARSISAEGLHNLRVLLEGQ

>Sobic.005G007900.1(SbPP2C35)

MGICCSKAAAGRELDDDDHGSGFPWMHDDVFHGHLWASAGVSVHTKQGWKGANQDAMTVSQDFAGHKGHIFCGVFDGHGPLGREVARYVRDTLPVKLASALKPKTADEDSSSDTLKLKPQEDDSSNDLKLTTEEDDSSNSLKLRTEEDPSSNTDLDSFDKSDSSSSSDDTSDESQLLSTWKNILVKTFEQVDGELRQHSGIDCICSGTTAVAAVRQGDHLIVANLGDSRAVLCTRDSKDRLIPVQLTTDLKPDLPSELARILNCKGRVFAMDDEPDVPRMWLPNQDAPGLAMARAFGDFCLKNHGLICTPEVYCRKLSEKDEFLVLATDGIWDVLSNKEVVKLVSSATDPSRAARQLIDRAVRAWRRKYPTSMVDDCAVVCLYLNRRASPGPGPDESLRVPGTGDDVKPFTGSSFRRALTSNGGGGEAEEGATVWRALEGVARANSVIRLPRLGRVLSWRRRSSSLDEDDGEERD

>Sobic.005G087800.1(SbPP2C36)

MLLHTVRFLCSVVAAFARLVRELRKAMATAACSPSPVAVLTAPAAVSPIGLIASTPLPKRRKTLAQFAVPENLLVAPPPPPAVVLPAKVSTSGDDDVSGVGMVEQQQEARQSLTKTKAAWAARRRPSRLVIPVADDAGEVAAGWAAAAAAPAKETDVEVEGDGFWVASRAGPRHAMEDAYAVVTDKNDADSQLAFYGVFDGHGGRAAVDFVSERLSKNVVSAVVAAAGTEARREASSEEDAVSAAIKAAYLATDSELLTQHQDASGGACAATAVVKGGDLYVAHLGDCRAVLSRGGAAAALTADHTCAREEERARIERQGGYVCRSGSGVWRVQGSLAVSRAFGDGALKQWVVAEPAVTRVPLVAGCEFLVMASDGLWDKVSNQEAVDVVSGSRATACRELVDMARCRGSRDDVTVMVVDLHRFVRED

>Sobic.005G167700.1(SbPP2C37)

MPLPVSGRRGLLILPILLLILDALPARLPRCAAESATCLAVYREGGAPAVFQSAHCPRWTLPPHDGQGDGGGKSSPMGCHVAADRGRRRSQEDRAVCALGIRIPFVEQMRIKEVDVGVVAIFDGHNGAEASEMASKLFLDYFLLHVYFLLDGIYSMLFRKSTGKLTYREITILNDLFNLYKDDQSNHGERSCWTSPAILDRSFHMEILKESLMRAVHDIDLTFSKEALQNNFESGSTATVVLIADGQIIAANVGDSKAFLCSQGYDSHHRNRKRRRKRNSISHEEFALANYDGPLYHARELTKDHHPDREDERSRVEAAGGYVLEWAGVYRVNGELALSRAIGDIPYKRYGVISTPELTGWQILSENDSFLIASSDGIFEKMTMQDVCDLMLHAKFRVQHNFGSSAITQHNLADYVVHVALQKGTTDNVAAVIVPLGSPGSTGTTLKDWSQFEEDLKTSISPVLNIPYRLEPDDGTSSAVIDMEYFKRSSIKFQRFLVEAKSKKLGCFYLSESLDEDMDYIFRVPEAYQRDEVHDFNHMPAETALYSDGNLEKYKDRHFCLYLGHQDGEMGQCNGPEAFANYFGLLDSLPHNGSKSNGSRSFGNTIDFRYKLKRRFDRGSYGEVWLAFHWNCSEHTDVHKEPPHFTTVSKSEMYNCTSSDTMSSDEDHVSGTVDGDLFILKRIMVERGNAAYLSGLREKYFGELFSNASRTLEGLSRTESSTISLDMQYNPDIRLERNLSATGEPLKHVARFIESFESESREIWLVYHNEGRSLSKLIYTAEETKLVTGNNNERVRHIQVLHPSKWWHWLRTTKAGQKQMQNLLWQLLMGLKACHDRNITHRDIKPENMIICFEDMETGKCLREVPPEAKQSNLNMRLIDFGSAIDDYTLKNLYGSGPTRSEQTFEYTPPEALLNSNWFQGSKSARLKYDIWSVGVVMLELMVGSPHVFQISDRTRILMDQRLKGWSEQTKELAYKLRSYMELCILVPGISSQHHGSGGPEQGQFGLASWKCSEESFAHQVKIRDPLKIGFPNLWALRLARQLLVWHPEDRLSVDEALKHPYFQEPT

>Sobic.006G018400.1(SbPP2C38)

MVMASSTGLNVPAGAGGDGVGNPTAAAAATEECRLRRRRRLETQGSGEAGPSWVRRLRPAAGSRFSPSSSSSSSAQEEEADPDVQEMELAPLPPPAAAGVGVGVVLQPQPEPEPEPEPEAPPVAAAVAAQRVWPLAFGSVTMAGRMRIMEDTVSLHPNLCYWPADGSPIHFFAVFDGHGGPHVSALCRERMHEFVAEELAKEGAAFLRRRQAAAAASSSSVAAAAPWSDQAEEERAWRAALMRSFRRVDAMAPLACACGRVTVTRPTPCGCPLSVSAASASAAAAAARRNNGIVGSTAVVAILVRGRLVVANCGDSRAVLCRGPQGTPPVPLSFDHKPNRPDERARIEAAGGQVVFINGYRVRGILAMSRAIGDRLLRREGVIAEPEITITERTVEDQCLILASDGLWDVMDNFIACYVARQCLEDGNPPPAAAAGGGGGGAPPAAGGVVGPQEDPRCVGAASLLGRLALGRETEDNISVIVVDLKHRQ

>Sobic.006G064100.1(SbPP2C39)

MVDVELFYKDTSDHSISSEEEDMLIRSCSNLNVSFGYHCDSSQSFSPENDHGNGISPKNIFGTNTMIGSRNGSFTCLSGAAISANFTLANTNICNGLIGEEILPELDSPNSFRKIVSSPSMSRLDSYSTSQGSPVSTESSIFEISKNLWRSSAPTTVSSNFLTNTEVKMAGGAAGEDRVQAVCSEKNGWLICGIYDGFNGRDAADFLAVTLYDNIVYYLYLLENRIKQQNGLYNSSETSLNGLKSELTLAMRNSENEDIKFSETFRAGVLNCLSTAVEQAENDFLCMVEQEMDDRPDLVSVGSCVLVVLLHGTDLCILNLGDSRAVLASMPYVQNGALKATQLTETHSLENPLEYQRLLADHPDDSSVVRGNKIKGKLKVTRAFGVGYLKQRKFNDALMGILRVRDLSSPPYVYTNPHTLSHKVTDDDLFVVLGSDGLFDFFSNDEVVWLVYQFMRDNPMGDPAKYLIEQLILKAAKEAALTAEELMRIPVGSRRKYHDDVTVIVIILGNAQRTMTASTSL

>Sobic.006G073100.1(SbPP2C40)

MALAVPVTLKTTEEGGNERFDYAVSAMQGYRPNMEDAHAIVLNLDAATGTSFFGVYDGQGGPAVSKYCARHLHTELLRHESFRDNLQTAIERTFLRMDEMMKDRSAGWELSGYGGNDNWKAYRKALRWSLLLPFFCQKPAYPGPENDGCTVCVVLIRGNQIIVGNAGDSRCVLSRNNVAVNLSTDFKPSLPATGHAVTFSERKCHRVDDGIAIAISLGDLLYKDNKDLCPQQQAITAFPEELTQDDQFLIIACNGIWDCQTRHQAVDFIRTQSNTVAYGMLKHGAQIAKFADKIRNTVTFVLDGSNYDVWEQYMMVMAHLRFWYICAHSTIQRNNTDRSQTGWD

>Sobic.006G098100.1(SbPP2C41)

MSSAASRSGRRRTGSVALGDLLRREASAERAALGGGGERERERERWPSVAAGQACRAKKGEDFALLKPACERRPGAPSTSFSAFALFDGHNGSAAAVYAKEHLLGNVLSCVPTDLSRDEWLAALPRALVAGFVKTDKDFQTRAHSSGTTVTLAIIDGSVVTVASVGDSRCVLEAEGSIYYLSADHRFDANEEEVGRVTECGGEVGRLNVVGGAEIGPLRCWPGGLCLSRSIGDQDVGEFIIPVPYVKQIKLSNAGGRLIISSDGVWDALTAEVAFRCARGLPPEAAAEQIVKEAVESKGLRDDTTCIVIDIIPPEKPKCTIESPKTPGKGLVLLKKFFLRKTASDSLSLADKDNYPEPDLVEEVFEDGCPSLSRRLNSEYPVRDMFKIFACAICQIELESGQGISIHEGLSKPGKLCPWDGPFLCHSCQEKKEAMEGKRHSRDSSSRNSGSSE

>Sobic.006G137000.3(SbPP2C42)

MGRPHSLILSLAQSLSLSLSLSYEDSPGSRISSFAMALAVPVTLKTTEEGGNERFDYAVSAMQGYRQNMEDAHAIVLNLDAATGTSFFGVYDGHGGPAVSKYCARHLHTELLRHESFRDNLQTAIEGTFLRMDEMMKDRSAGWELSGYGGNDNWKAYKKALRWSLLLPFFCQKPDYPGPENDGCTACVVLMRGNQIIVGNAGDSRCVLSRNNVAVTLSTDFKPSLDAERARIVAAGHEVTFSERGNMHRIDDGIAVSRSLGDLLYKDKHDLDPQQQAITAFPEVRTEQITQDDQFLIIACDGIWDCLSSQQAVDFIRIYLNSDVGLAFICEALLGHCLSHPRGRDNMTVVLVRFKTPGACQVRLPRPINQAAAARAAAEQERAAAEQERAAAEQEQAAEEQERAATEQAAATRAASTSAPSSPSFRRPKTEESPKSSKSSEL

>Sobic.006G192200.4(SbPP2C43)

MWPWLERIASACWDRVRRYGLTRKGEDDGGDGGEGDAGAVDELLWSRDLARHAAGEFSFAVVQANDVLEDQSQVETAAAATFVGVYDGHGGAEASRFISNHLSAHIVRLAQEHGTMSEDVVRKAFSATEEGFLSLVRRTHLIKPAMATIGSCCLVGIIWRGTLYLANLGDSRAVVGCLNGSNRIVAEQLTRDHNASMEEIRQELRTLHPDDSQIVVLKNGVWRIKGIIQVSRSIGDAYLKKREFAADPSTARFHLSEPLRRPVLTSEPSVCSRVLSSQDRFLIFASDGLWEHLSNQQAVEMVHNNPREGIARRLVQAALKEAARKREMRYGDIKKLDKGVRRYIHDDITVVVVFVDHELRSEDSASTSVPELSVRGFIDAGGRSSFSGMNDIT

>Sobic.006G209400.1(SbPP2C44)

MVGRMERQTASSSASCSPSAAAAASSSSSSVCGGKKRPDILNMIRNAACLNSSSTDTGKGRSKLSTNKVAHGFHLVEGKSGHDMEDYHVAEYKYVKNHELGLFAIFDGHLGDKVPCYLKANLFSNIMKEPLFWSSPQEAIKNAYCSTNKFILENAKQLGPGGSTAVTAIVVDGKDMWIANVGDSRAVVCEKGAANQLTVDHEPHTTNERQRIEKHGGFVTTFPGDVPRVNGQLAVARAFGDQSLKAHLSSEPDIRHVPISSNIEFVILASDGLWKVMKNQEAVDLVKSIKDPQAAAKRLTTEALARKSKDDISCIVIRFRC

>Sobic.006G225600.1(SbPP2C45)

MEKFCCFGAGCSDFTGHGSTASGKGKVCHGQVKVSYGYNLVRGMTNHPMEDYHFVDLVEVKGNELGLFAIYDGHLGDTVPAYLQKNLFTNILNEEEFWTHTDRAITKAYEKTDQAILSHTPDLGQGGSTAVTAILVNGRKLWVANVGDSRAVLLKGGEPIQMSIDHDPNVERSVIENRGGFVSNMPGDVPRVCGQLAVSRAFGDRNLKSLLRSEPDIKVEDIDHTAELLVLASDGLWKVMNNQEVVDLAKRYKDPYAAAKQLTAEAQKRESKDDISCIVVRFKA

>Sobic.006G249700.1(SbPP2C46)

MGYLSSVIGHPTDGSSVSGGGLSQNGKFSYGYASSPGKRASMEDFYETKIDCVDGQIIGLFGVFDGHGGAKVAEYVKENLFNNLVSHPKFMSDTKVAIDDAYKSTDSEFLESDSSQNQCGSTASTAVLVGDRLFVANVGDSRAIICREGNAIPVSKDHKPDQTDERQRIEEAGGFVMWAGTWRVGGVLAVSRAFGDKLLKQYVVVDPEIREEAVDDTLEFLILASDGLWDVVSNEEAVAMTRSIQDPEEAAKKLLQEAYKRESSDNITCVVVRFLRGQGSSGYA

>Sobic.007G078000.4(SbPP2C47)

MMGGGRAADSQPASCSSSSSSSSSPSGPSTGAPRAHRSKRRPDILNMLMSAACLSSSSSDTGKGQSKLSSNKVTHGFHLVEGRSGHDMEDYHVAEYRYENDHELGLFAIYDGHLGDSVASYLKVHLFDNILKEPLFWSDPQEAIKNAYSSTNKYILENTKQLGPGGSTAVTAIVVDGTDMWIANIGDSRAVVCERGTASQLTVDHEPHTTNERKRIEKQGGFVSTFPGDVPRVNGQLAVARAFGDQSLKAHLSSEPDVKHIKINSSIEFVILASDGLWKVMKNQEAVDLVKSTKDPQTAAKRLTSEALGRMSKDDISCIVIRFRC

>Sobic.007G216600.1(SbPP2C48)

MIQKLHKTCKLQASSKPYSHITVCSQRTQHNSQAGSSSYHPHPVLHRVASFLLAAMGSCLSSEAPAAGAGPAVWRKRRRGSREGAAGAGAGAGGGKKLPGGGGEMTEDELARVSGRMCGNGASAVACLHTQQGRKGTNQDAMVVWESFDSSESIFCGVFDGHGPYGHFVAKKVRDSLPVKLRTQWQTSANGGSSPHQNGSISGSINSEETGSIVDDEWGDGDDTEKLPEMFLPLKKSYLKAFKLMDKELKLHPTVDCFCSGSTAVTLVKQGLDLVIGNLGDSRAIMGTRDASNNLTALQLTVDLKPNLPREAARIQQCKGRVFALQDEPEVARVWLPNNDSPGLAMARAFGDFCLKDYGLISVPEISYRRLTEKDEFIILATDGVWDVLSNKEAVDIVASAPSRATAARALVDCAVRSWRLKFPTSKSDDCAVVCLFLDHEKSADLVQESEPSVETAEPTGEEVSTQDASAEVDEEIADASVHVSSAEHSAEATLEHSTTLREVDEIVPVDETPILKEPERCGSARSLADCISTNEEEEWSALEGVTRVNSLLNLPRILSGDKRSTSWRKRR

>Sobic.008G008500.1(SbPP2C49)

MGICCSKAAAGRELDDDQQGLGFPWMHDDLFHTHLWASAGVSVHTKQGWKGANQDAMTVSQDFAGRKGHIFCGVFDGHGPHGREVARHVRDTLPVKLASVLKPKTGDEDSASDSLKHKPEEDDSSNNLKLKPGEADSSNNLKLKPEEDDSSNSLKLRTEEDHSSNGSKLRTEEDPSSNTDLDSFDKSDSSSSSDDTSDESQLLSTWKNIFVKAFEQVDEELRQHSGIDCICSGTTAVAAVRQGDHLIVANLGDSRAVLCTRDSKDRLIPVQLTTDLKPDLPSELARILSCKGRVFAMDDEPDVPRMWLPDQDAPGLAMARAFGDFCLKNHGLICTPEVYCRKLSEKDEFLVLATDGIWDVLSNKEVVKLVSSSTDPSKAARQLIDRAVRAWRRKYPTSMVDDCAVVCLYLNRRASPGSDESLRVPGIGEDVKPPAVPFTGSSFRRALSSGGGEASSEEGATVWRALEGVARANSVIRLPRLGRVLSWRRRSSSLDEDDGEERE

>Sobic.009G032400.1(SbPP2C50)

MVAEAEVMHQTPVPVLEVQYHRCVTTKGVEDVVGMSAAAAAAVAAPAEVGVEVEVEVEVEVEVAVEVPLMGLEQPDAAPSVSMDVLQFVPTIRSGSFADIGPRRYMEDEHIRIDDLSAHLGSLLVCPLPSAFYGVFDGHGGPDAAAYMKRHAMRFLFEDSEFPQASQVDEMYVQSVESSVRRAFLQADLALADDLDISRSSGTTALTALVFGRQLLVANAGDCRAVLCRKGVAMEMSRDHRANYVEECERVAASGGYIEDGYLNGVLSVTRALGDWDMKTPDASVSPLIAEPEFRQATLGEDDEFLIMGCDGIWDVMTSQHAVSLVRRGLRQHDDPARCARELVMEAKRLETADNLTVIVVCFASEMGSQQQEQPVRPRSCKGLSTEALCNLRSWLETDHR

>Sobic.009G109700.1(SbPP2C51)

MRQISSLLQGLARSLSVGREKDRKGDGGAGGDGGKTAMPAVLRTSGTLWGEGSETFAAVCSRRGEKGINQDCSIVWEGFGCQEDTIFCGIFDGHGPWGHYVAKAVRDSLPPSLLCHWQEALALASLIDGEKKRLSDCQLDLWKQSYVAACAAVDDELRRSRRFDAVQSGCTALSIVKQGDLMVVANVGDSRAVLGTTSDDGGGAIAAVQLTVDFKPNLPQEKERIRRCNGQVYCLADEPGVHRVWQPNRESPGLAMSRAFGDYCVKDYGVISVPEVTQRRITTGDQFVILATDGVWDVLSNDEAVRIVAGTPDREKAAKRLVECAVHAWRRRRRGFAVDDCSAICLFFHSPSS

>Sobic.009G161800.1(SbPP2C52)

MAEICCEETKSTPPASASAITALARGRPRVEMAVAEAACRSSSSSTPAADEGAGGGGGASATRCGKRRRVACAAPVRSCQRSRTTSGFGSTWWWPRYGVTSVCGLRREMEDAVSIRPDFLRGAADPSSGKHHFYGVFDGHGCCHVARMCQDRMHALVADEYKKAGSGNSATAAAAEPAWEEVMKKVFARMDGEAATRAASRSSNGSIACRCELQKPTRCDHAGSTAVVAVVSPTSVVVANAGDSRAVLSRAGVPVPLSVDHKPDRQDELERIQEAGGRVIYWDGARVLGVLAMSRAIGERRAAPRAFVRNPSELSVQTNIFGPPCFSLKLFGRAGDGYLKPFVTAEPEVAVTERTDDDECLILASDGLWDVVTNEMACDVVRACFRSNGPPSPPAARPNGVLPPPAAGAGREGDDGAASPVKGVDKADSDRACSEAALLLAKLALARRSTDNVSVVVVDLRRRC

>Sobic.009G213000.1(SbPP2C53)

MAATAAICVEDEATCAAAECAPGVEKLDLAAAGAGADGKAGGGKRSVYLMDCAPVWGCASTRGRSAEMEDACAAFPRFADVPVRLLARRRDLDGLGLDADALRLPAHLFGVFDGHGGAEVANYCRERLQVLLRQELRLLGKDLGETSEVEMKEHWDDLFTRCFQRLDDEVSGQASRLVGGVQESRPVAAENVGSTAVVAVVCSSHVVVANCGDSRVVLCRGKEPVELSIDHKPDRKDERARIEALGGKVIQWNGYRVSGILAMSRSIGDRYLKPFVIPKPEVTVFPRAKDDDCLILASDGLWDVIPNEEACKVARRQIQLWHKNNGVASSLCDEGDESTDPAAQAAADYLMRLALKKGTEDNITVIVVDLKPRKKLKSNS

>Sobic.009G238600.1(SbPP2C54)

MRETSAARDEGGPAVCKAVAGGNVDRKGGASARRRRRLELRRLGRATEGSPVPAKRIRSGSHRSSSSDSSLETAAAGCWPPRLSHGAVSVIGRRREMEDAFAIALSFLASDPSSPGAKDEQEQDFFAVYDGHGGARVAEACRERMHVVLAEELGLRRGVGSDDLRWEEAMAASFARVDGEVTGGFSPPPKPPPQQTAADAADTNAGAGNLPYRTVGSTAVVAVVGQRRIVVANCGDSRAVLSRGGVAVPLSTDHKPDRPDEMQRVEAAGGRVINWNGYRVLGVLATSRSIGDYYLKPYVSAEPEVTVVERTEQDEFLILASDGLWDVVSNEMACKIARNCLNGRAASKFPGSVAGRTAADAAALLTELAMSRGSRDNISVVVVELQRLKTSPGAA

>Sobic.009G250900.1(SbPP2C55)

MVCDGAVKDQDQETAAASTASAASVVAASEASPAPVVVVVSARPSARSSPHDKRLGVRHPLKHRRFRAGGKMMVEPGGVPPAHAVPEGEEEEEEEEEGASEVEEEELVEAEAEQASSAGTAMRGTEVEVSSAPAAGVQEMEVEVEGGEMEVKYGEMEVSPEPAVAVGGTELEAHPDEEDEVSSISVARGEKKQEAASPPPAASAVLAVEAPREMGQYGERAEKERRDKERERQKERERVDEVGYMSGGWKSVDGTLNCGYSSFRGKRASMEDFYDIKSSKIDDKQINLFGIFDGHGGSRAAEYLKEHLFENLMKHPEFMTNTKLAISETYRKTDSEFLDAERNTHRDDGSTASTAVLVGDHLYVANVGDSRAVISKAGKAIALSEDHKPNRSDERKRIESAGGIVMWAGTWRVGGVLAMSRAFGNRLLKQFVIADPEIQEQEINDELEFLIIASDGLWDVVPNEDAVTLVKMEEEPEAAARKLTETAFSRGSGDNITCIVVKFQHDKPGSSGGGDPPSSPPGDKS

>Sobic.009G255900.1(SbPP2C56)

MDDLTAGGLELFAAVADFIDDSAPPAAAMPPQQPEPDSAPGSPCSVASDCSSVATADFEGFADLGSALVLDDLVASASTSLPASGPRASAAAAPARSVFALDCVPRWGLESVCGRRPEMEDAARVLPTFFHVPLWMLAGDAPVDGLDRASFRLPTHFFGVYDGHGGLQVANYCRERIHKVLAEELTKAQEAASDADLSALDPNNTHKHWEKAFVDCFSRVDAEVGGNAATQGKPVAPDTVGSTAVAALVCSSHVIVANCGDSRAVLCRGKQPLTLSVDHKPNREDEYARIEAQGGKVINWNGYRVLGVLAMSRSIGDRYLKPYVIPVPEVTIVARAKDDECLIIASDGLWDVMSNEEVCDAARKRILLWHKKNADASSSAQRSGDSADEAAQAAAEYLSKLALQKGSKDNITVIVVDLKSHRKFKSRT

>Sobic.010G062500.1(SbPP2C57)

MCVEEPEDAERLGFGDAGVEEPAKFPLVQMERVCENTTSADFRQKLSNFVPVIRSGDWSHIGGRQYMEDTHVCIPDLAKNFGFPSLDNEVVSFYGVFDGHGGKDAAHFVRDNLPRVIVEDSDFPLQLEKVVRKSFMQTDCQFAETCSLHRATSSGTTALTAMIFGRSLLVANAGDCRAVLSRCGTAIEMSMDHRPCSLSEKLRVESLGGYVDDGYLNGLLGVTRALGDWHLEGMKEVGEPGGPLSADPELKMITLTKDDEFLIIGSDGIWDVFSNQNSVDFARKRLQEHNDVKLCCKEIVEEAIRRGATDNLTAVLVTFHLEAPPQIKVDRPGRVARSISAEGLNSLRILLGRQ

>Sobic.010G247600.1(SbPP2C58)

MGFAEKGSPPVTGGGFSENGKFSYGYASSPGKRSSMEDFHETRVDGVDGETVGLFGVFDGHGGARAAEFVKQNLFTNLIKHPKFFSDTKSAIAETYTHTDSELLKADTSHHRDAGSTASTAILVGDRLLVANVGDSRAVICRGGDAIAVSRDHKPDQTDERQRIEEAGGFVMWAGTWRVGGVLAVSRAFGDKLLKQYVVADPEIKEEVVDSSLEFLILASDGLWDVVTNEEAVAMAKPILDSEQAAKTLLAEASQRGSADNITCVVVRFLEQQNGLAGRATNDQAS
